# Thermal Degradation Characteristic and Flame Retardancy of Polylactide-Based Nanobiocomposites

**DOI:** 10.3390/molecules23102648

**Published:** 2018-10-16

**Authors:** Kuruma Malkappa, Jayita Bandyopadhyay, Suprakas Sinha Ray

**Affiliations:** 1 DST-CSIR National Centre for Nanostructured Materials, Council for Scientific and Industrial Research, Pretoria 0001, South Africa; MKuruka@csir.co.za (K.M.); jbandyopadhyay@csir.co.za (J.B.); 2 Department of Applied Chemistry, University of Johannesburg, Doornfontein 2028, Johannesburg, South Africa

**Keywords:** polylactide nanocomposites, intumescent flame retardancy, synergistic effect

## Abstract

Polylactide (PLA) is one of the most widely used organic bio-degradable polymers. However, it has poor flame retardancy characteristics. To address this disadvantage, we performed melt-blending of PLA with intumescent flame retardants (IFRs; melamine phosphate and pentaerythritol) in the presence of organically modified montmorillonite (OMMT), which resulted in nanobiocomposites with excellent intumescent char formation and improved flame retardant characteristics. Triphenyl benzyl phosphonium (OMMT-1)- and tributyl hexadecyl phosphonium (OMMT-2)-modified MMTs were used in this study. Thermogravimetric analysis in combination with Fourier transform infrared spectroscopy showed that these nanocomposites release a smaller amount of toxic gases during thermal degradation than unmodified PLA. Melt-rheological behaviors supported the conclusions drawn from the cone calorimeter data and char structure of the various nanobiocomposites. Moreover, the characteristic of the surfactant used for the modification of MMT played a crucial role in controlling the fire properties of the composites. For example, the nanocomposite containing 5 wt.% OMMT-1 showed significantly improved fire properties with a 47% and 68% decrease in peak heat and total heat release rates, respectively, as compared with those of unmodified PLA. In summary, melt-blending of PLA, IFR, and OMMT has potential in the development of high-performance PLA-based sustainable materials.

## 1. Introduction

Polylactide (PLA) is one of the most promising sustainable polymers, owing to its excellent physical properties and mechanical capabilities, low cost, large production volume, and its transparency [1,2]. Because of these characteristics, PLA can be used in numerous applications, including paper coatings, fibers, films, foams, molded parts, and packaging [3,4,5]. However, some inherent properties of PLA, such as its low thermal stability and high flammability, limit its expansion into diverse applications, such as flexible electronics, constructions, and engineered plastic materials. Efforts have been made to improve the flammability characteristic of PLA by the introduction of P- and N-containing flame retardant components; nanomaterials such as montmorillonite (MMT) [6,7], graphene oxide (GO) [8], zinc oxide (ZnO) [9], molybdenum sulphide (MoS_2_) nanosheets [10], and layered double hydroxide (LDH) [11]; and various synergistic combinations [12,13,14].

MMT is a type of multilayer aluminosilicate smectite clay with inorganic nano-sized particulates and widely used as a flame retardant (FR) for polymer nanocomposites because of its low cost, abundance, and excellent FR performance [15,16]. The MMT mainly inhibits the release of toxicants, heat, and smoke from the polymer composites during combustion [6]. However, compared with those of pristine MMTs, OMMTs show greatly improved FR properties. Pristine MMTs often exist in polymer matrices at the micro scale because they are hydrophilic in nature and do not have thermodynamically favorable interactions with the hydrophobic polymer matrices. Hence, the silicate platelets easily agglomerate or separate in the polymer matrix and it is difficult to achieve the required properties of the polymer nanocomposites (PNCs). To render MMTs compatible with hydrophobic polymer matrices, one must convert the normally hydrophilic surface to a hydrophobic one (commonly known as OMMTs), making the intercalation of polymer chains possible. Generally, this can be done by ion-exchange reactions with cationic surfactants, which lower the surface energy of two-dimensional silicate layers and improve the wetting characteristic of the polymer matrix, and result in a high silicate interlayer spacing. Some OMMTs are effective when added to various polymers, and significantly enhance the FR properties of the PNCs [17]. To pass a fire test, a polymer combined with OMMT must also be combined with other types of FRs because MMT alone cannot improve the fire properties of PNCs necessary for specific applications.

Melamine phosphate (MP), derived from the reaction between melamine (MEL) and phosphoric acid, is typically used as an intumescent FR (IFR), alone or combined with pentaerythritol (PER) in many applications, such as FR coatings and plastics. The typical IFR system comprises three types of components: an acid source, a char forming agent, and a blowing agent [18]. MP provides two of these three components, an acid and gas source, and MP can be an excellent FR for carbon sources containing polymers [19]. Casetta et al. [20] studied the efficiency of IFR formulations with PLA, which contained ammonium polyphosphate (APP), PER, lignin, and starch. The results showed that the FR activity of the PLA composites was greatly improved with a high loading of 40 wt.%. Using a combination of microencapsulated ammonium polyphosphate (m-APP) and lignin as the IFR, a PLA/IFR composite was prepared, and it displayed excellent FR activity with a high loading of 23 wt.% [21]. Similarly, another FR system, using a PLA matrix consisting of polyhedral oligomeric silsesquioxane/m-APP and MMT/APP was studied [12,22]. The IFR contained a mixture of MP and PER used for polyolefins (such as polypropylene, PP), and the obtained composite exhibited excellent FR activity. It was also reported that the PP/MP composite system containing PER or its derivative compounds (DPER and TPER) displayed efficient FR activity through the formation of highly compact intumescent char, resulting in lower smoke and heat release rates, a high LOI value, and a UL-94 rating, compared with those of the PP composite containing MP alone [23]. There are various factors that influence the char formation mechanism during combustion, such as composition and ratios of IFR, their decomposition behavior, and the characteristics of the polymer resins [24]. The advantage of these IFR compositions is that the presence of PER inhibits the melamine sublimation and accelerates FR activity in the condensed phase with improved char residue and strength. However, to the best of our knowledge, there is no report on a PLA/IFR/clay composite system.

In this study, we used MP/PER as an IFR to inhibit melamine sublimation. We prepared PLA/IFR/OMMT nanocomposites using two different types of OMMTs and different weight-percentage loadings. The resulting nanocomposite FR activity, toxic gas evolution, and thermal stability were characterized using thermogravimetric analyzer (TGA), cone calorimetry, and TGA hyphenated with Fourier transform infrared spectroscopy (TG-FTIR). We also studied the char morphologies of the char residues after cone calorimetry tests, and their FR behavior is thoroughly discussed.

## 2. Materials and Methods

### 2.1. Materials

**Figure 1:**
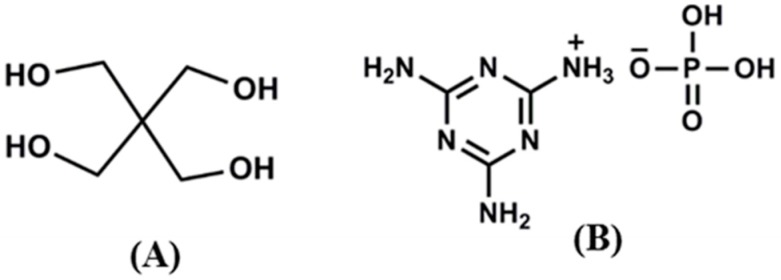
Molecular structure of (**A**) pentaerytritol and (**B**) melamine phosphate.

PLA resin (7032D) with a specific gravity of 1.24 and an MFI (melt-flow index) of 13.81 g/10 min (190 °C, load = 2.16 kg) was obtained from Nature Works LLC (Minnetonka, MN, USA). The pristine sodium montmorillonite (MMT) used in this study is Cloisite® Na, purchased from Southern Clay Products (Gonzales, TX, USA). According to the supplier the cation exchange capacity (CEC) value of MMT is 92.6 meq/100 g of clay. Other chemicals, such melamine, phosphoric acid, triphenyl benzyl phosphonium chloride, tributyl hexadecyl phosphonium bromide, hydrochloric acid, silver nitrate, and pentaerytritol (PER) (Figure 1A, molecular structure) were purchased from Sigma Aldrich (Johannesburg, South Africa) and were used without further purification.

### 2.2. Synthesis of Melamine Phosphate

Melamine phosphate was synthesized based on a method reported in literature [25]. First, 15 g of melamine was dissolved in 200 mL of water and stirred for 1 h at 95 °C. Phosphoric acid (85 wt.%, 4 g) was dissolved in water separately, then this solution was slowly added to the reaction mixture, left for 3 h with continuous stirring, and allowed to cool and crystalize. Next, we separated melamine phosphate (MP) (Figure 1B, molecular structure) crystals by filtration and kept them in an oven at 100 °C for complete drying.

### 2.3. Surface Modification of MMT

**Scheme 1:**
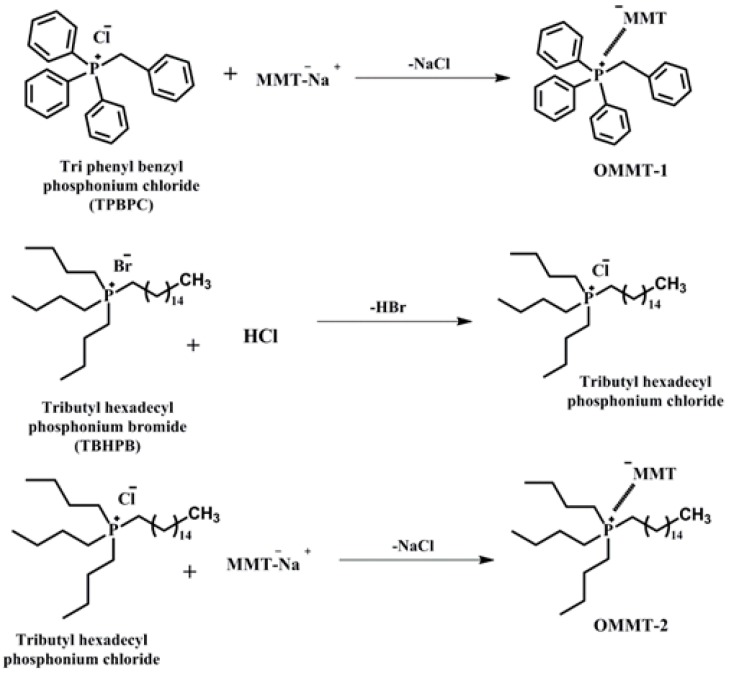
Molecular structure of triphenyl benzyl phosphonium chloride and tributyl hexadecyl phosphonium bromide surfactants and the preparation of organically-modified montmorillonite from Na-montmorillonite. Drawings are not based on proper scale.

The MMT was organically modified using two different types of phosphonium derivatives, following previously reported methods [26,27,28]. Briefly, the procedure is as follows: the MMT (5 g) was dispersed in water and stirred at 90 °C for 12 h to improve the silicate layer dispersion. Next, the required quantity (6 mmol) of triphenyl benzyl phosphonium chloride surfactant was dissolved in water separately and then slowly added to the MMT mixture, followed by continuous stirring for 24 h to achieve complete intercalation of triphenyl benzyl phosphonium cations into the clay galleries. Then, the mixture was washed with water and ethanol until the chorine anions were absent in the filtrate in a chorine test with AgNO_3_ solution, and the resultant triphenyl benzyl phosphonium modified MMT was kept in oven at 100 °C for complete drying to prepare the nanocomposites. This triphenyl benzyl phosphonium modified MMT was designated as OMMT-1. To remove bromine from tributyl hexadecyl phosphonium bromide, after completely dissolving in water, we added HCl and stirred for 1 h to complete the exchange of bromine ions with chlorine ions, because of the low ion exchange capacity of bromine compared with that of chlorine, as shown in Scheme 1. Then, the above procedure was followed to prepare tributyl hexadecyl phosphonium-modified MMT and the modified MMT was designated as OMMT-2.

### 2.4. Nanocomposite Processing

A co-rotating twin-screw extruder with L/D of 40 (Process 11, Thermo Scientific, Waltham, MA., USA) was used for nanocomposite processing. A screw speed of 200 rpm was used and the temperature profile for extruder was 160, 170, 190, 190, 190, and 170 °C, and that for the die was 190 °C. To avoid thermal degradation of the PLA matrix, there was a constant flow nitrogen gas in extruder. The extruded samples were collected via a water bath, pelletized, and then dried at 100 °C for overnight in a fan oven. Table 1 summarizes the composition and abbreviation of various samples used in this study. Finally, dried nanocomposite pellets were compression molded at 190 °C to prepare samples for various characterizations.

### 2.5. Characterization

Small-angle X-ray scattering (SAXS) experiments were performed using a line-collimated SAXSess system (Anton Paar, Graz, Austria) operated at 40 kV and 50 mA; Cu K_α_ radiation with a wavelength of 0.1542 nm (PAN analytical X-ray source, Eindhoven, The Netherlands) was used. A sample holder (paste-cell) for powder samples was used to examine MMT and OMMTs. A transmission electron microscopy (TEM) instrument, JEOL JEM 2100 HRTEM (JEOL, Tokyo, Japan) operating at 200 kV, was used to evaluate the morphology of nanocomposite microtome samples using a carbon-coated grid. Images were captured using a TEM camera (Ultrascan, Gatan, Pleasanton, CA, USA) and Digital Micrograph software (Gatan, Pleasanton, CA, USA). TGA analysis was performed using a TA instrument (model TGA Q500, New Castle, DE, USA) from 30 to 800 °C with heating rate of 20 °C/min under a nitrogen and oxygen gas atmosphere. After a cone calorimetry test, the microstructure of the char residues was recorded using Scanning Electron Microscopy (SEM, AURIGA Crossbeam workstation from Carl Zeiss, Germany) operated at 3 kV. To identify the degradable volatile components of the neat PLA and PLA/OMMT nanocomposites, TGA-FTIR was carried out using a PerkinElmer Pyris 1 TGA connected to a Nicolet IS50 Spectrometer (PerkinElmer, Waltham, MA, USA) with a temperature range of 50 to 800 °C and a heating rate of 20 °C/min under a nitrogen atmosphere. About 20 mg of each sample was tested. The fire performance of all the PLA/IFR/OMMT nanocomposite samples and PLA were evaluated by iCone Calorimetry (Fire Testing Technology, East Grin stead, UK according to ISO 5660). Calorimetry test samples with the dimensions of 100 mm × 100 mm × 3 mm were prepared; the samples were wrapped in aluminum foil, leaving the top surface open, and were exposed to radiant cone heating with a 25 kW/m^2^ heat flux. Melt-rheological analysis was performed using a Physica MCR 501 Rheometer (Anton Paar, Graz, Austria) equipped with 25 mm parallel plates. Time sweep tests were carried out under a normal atmosphere and strain amplitude of 0.5% (linear region) within a 20 min period at 190 °C.

## 3. Results and Discussion

### 3.1. Structural and Morphological Analysis

**Figure 2:**
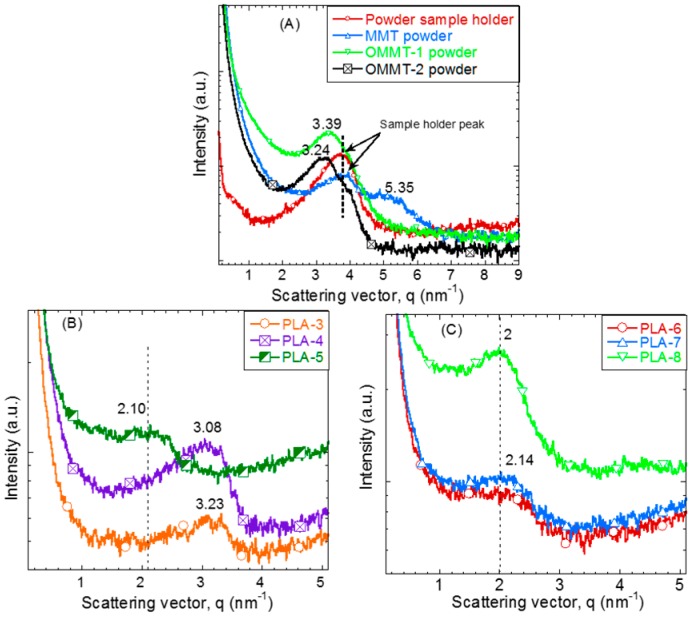
XRD patterns of PLA and different surface-modified OMMT-containing PLA nanocomposites with different weight percentages. (**A**) OMMTs, (**B**) PLA/IFR/OMMT-1 nanocomposites, and (**C**) PLA/IFR/OMMT-2 nanocomposites.

SAXS patterns can generally be used for structural analysis of PNCs, including mode of dispersion (intercalation or exfoliation) of the silicate layers in the polymer matrix. The scattering patterns of MMT (Na^+^-MMT), two OMMTs, and PLA/IFR/OMMT nanocomposites with different weight percentages of IFR and OMMT loadings are shown in Figure 2. The peak at *q* = 5.35 nm^−1^ (*d*_(001)_ = 1.17 nm) corresponds to Na^+^-MMT, and after surface modification with different phosphonium derivatives, OMMT-1 and OMMT-2, the peak shifts to lower angles, *q* = 3.39 nm^−1^ (*d*_(001)_ = 1.85 nm) and 3.24 nm^−1^ (*d*_(001)_ = 1.94 nm), respectively, as shown in Figure 2A. This indicates that intercalation of phosphonium compounds into the clay galleries occurs, resulting in an increase in the *d*_(001)_-spacing, which is in good agreement with previous reports [27,29]. After addition of the OMMT-1 with varying the weight percentages to the PLA, significant changes in the PLA/IFR/OMMT-1 composites were observed in the lower scattering vector region with increasing OMMT-1 concentration, which indicates a formation of highly intercalated nanocomposite structure, as shown in Figure 2B. For the nanocomposites containing 1 and 3 wt.% of OMMT-1 (samples PLA-3 and PLA-4, respectively), the characteristic OMMT-1 peak shifted to a lower scattering vector, *q* = 3.23 (*d*_(001)_ = 1.94 nm) and 3.08 (*d*_(001)_ = 2.04 nm), respectively, which may be due to further intercalation of the PLA chains in the two-dimensional silicate galleries. In the nanocomposite containing 5 wt.% OMMT-1 (PLA-5), the characteristic peak of OMMT-1 was significantly shifted to lower side with a broader peak, appeared at *q* = 2.10 nm^−1^ (*d*_(001)_ = 2.99 nm). Such a result indicates high-level of intercalation occurred in presence of MP and PLA chains enter into the silicate layers. On the other hand, Figure 2C illustrates scattering patterns for different weight percentages of OMMT-2 containing PLA composites (PLA-6 to PLA-8). In all these nanocomposites, an intercalated nanocomposite structure was observed and in peak not much shifted lower side as compared to earlier.

**Figure 3:**
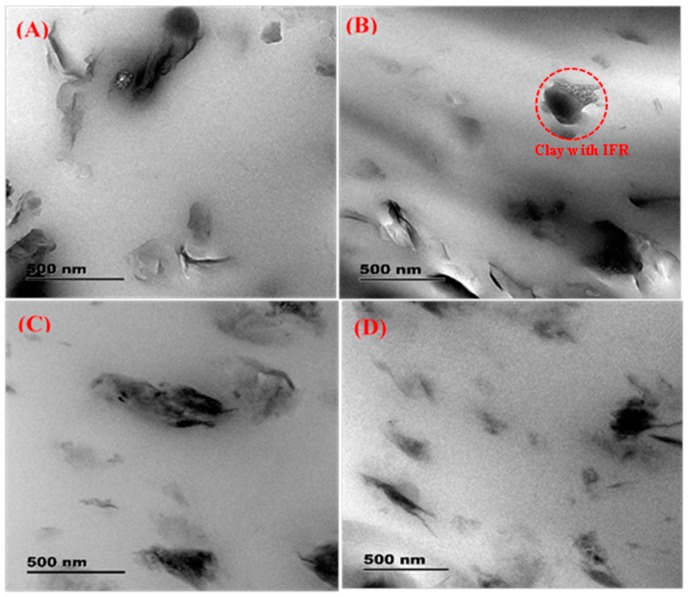
TEM images of PLA/clay nanocomposites. (**A**) PLA-3, (**B**) PLA-5, (**C**) PLA-6, and (**D**) PLA-8.

The TEM images of the representative nanocomposites PLA-5 and PLA-8 are shown in Figure 3 at two different magnifications. The images show dark strips in some regions, either related to MMT and IFR agglomeration or partially exposed surfaces of intercalated silicate layers, and the thick, light dark lines indicate some degree agglomeration of intercalated silicate layers in the PLA matrix. Moreover, TEM images show that agglomerated and intercalated silicate layers are well distributed in the PLA matrix and the disordered nature of intercalation, which agrees with the presence of lower angle (001) characteristic peaks in the scattering patterns of PLA-5 and PLA-8.

The different dispersion characteristics of PLA-5 and PLA-8 nanocomposites can be explained on the basis of flow behavior during processing. Detailed melt-state flow behavior of various samples can be found in Section 3.5. It can be seen that the transient viscosity of PLA-8 at 190 °C is higher than that of PLA-5 and remains constant throughout the experiment. However, in the case of PLA-5, the viscosity increases with time. Such increase in viscosity results in increase shear-stress during processing, which facilitates the peeling of silicate layers from the clay-stacks as shown in PLA-3 (Figure 3A) and PLA-5 (Figure 3B). On the other hand, initial higher viscosity, and hence the stress, breaks down clay-stacks into small clay-stacks which hinders the peeling mechanism. For this reason, a large number of small intercalated clay-stacks observed in the TEM image of PLA-6 (Figure 3C) and PLA-8 (Figure 3D).

### 3.2. Thermal Degradation Characteristic

**Figure 4:**
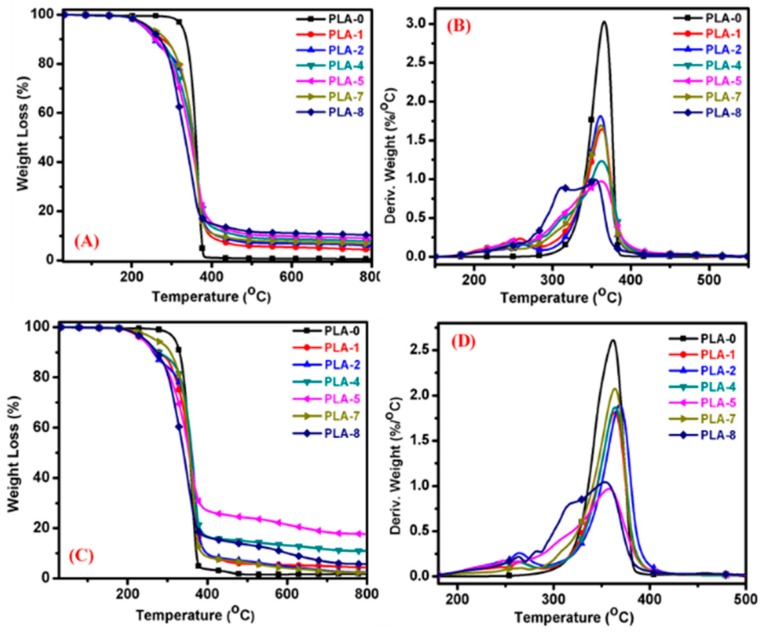
TGA and derivative plots of PLA/Clay nanocomposites with different surface-modified clays and compositions. (**A**,**B**) under nitrogen atmosphere and (**C**,**D**) under air atmosphere.

The thermal degradation characteristic of the nanocomposites was studied using TGA and the relevant thermal degradation data includes *T*_deg_, defined as the temperature at which degradation started, *T*_max_, defined as the temperature at which maximum weight loss occurred, and the percentage of char residue present at 800 °C in different environmental conditions, such as under an air and nitrogen atmosphere. The TGA plots of neat PLA and various nanocomposite samples are shown in Figure 4 and the characteristic data calculated from various TGA thermograms are summarized in Table 2. With the addition of IFR and IFR/OMMT to the PLA, the initial degradation temperature gradually decreased from PLA to PLA/IFR and then to PLA/IFR/OMMT nanocomposites due to the presence of IFR and IFR/OMMT, which accelerate the polymer degradation to fast char formation that acts as thermal insulation at high temperatures to protect the underlying polymer from further degradation and improve the char residue. However, the combination of IFR with OMMT further increased the char residue because of the high phosphorous content. In this thermal degradation, first MP started to decompose and release melamine pyrophosphate and polyphosphates, which further condensed the polymer chains to form char [30]. In addition, the OMMT-1-containing nanocomposites, PLA-4 and PLA-5, improved the char residue, compared with that containing OMMT-2, PLA-7 and PLA-8, because aromatic phosphonium compounds are more resistant to thermal degradation than aliphatic phosphonium compounds. Overall, it was observed that in both the nitrogen and air atmospheres, the thermal degradation phenomenon were similar (Figure 4A,C). However, compared with those of the N_2_ atmosphere, the air atmosphere improved the early polymer degradation and largely increased the char residue from PLA to IFR/OMMT-1 containing PLA nanocomposites. The relevant values are included in Table 2. Because of the catalytic activity of the MMT during thermal degradation in the presence of oxygen, the surfactant triphenyl phosphonium oxidizes to form a stable triphenyl phosphine and phosphite oxide, which may create a protective barrier on the surface of the polymer to delay the polymer degradation, even at high temperatures [31]. Also, it clearly indicates that PLA-5 produced a high char residue of about 18.5% at 800 °C. Owing to possible synergistic effects, it has strong thermal oxidative behavior even at high temperatures. Figure 4B,D shows the TGA derivative plots of nanocomposites and the addition of IFR, pristine MMT, and OMMTs to the PLA. The degradation behavior obviously changed from high peak intensity to low peak intensity. With increasing loading of IFR/OMMT to the PLA, the *T*_max_ and α_max_ values were slightly decreased. The decreasing value of α_max_ with decreasing peak intensity indicates that the presence of IFR/OMMT promoted the PLA degradation at lower temperatures to form thermally insulating char faster, which can prevent further polymer degradation. Therefore, the peak intensity of PLA-5 was lower than that of PLA-8. Also, PLA-8 contained a broad degradation peak, clearly visible in Figure 4B,D, indicating higher degradation. Moreover, the residual char yields at 800 °C for PLA-5 and PLA-8 were much higher than the PLA-0, as shown in Table 2. All these results suggest that rearrangement reactions occur in between the MMT surface modified by surfactants and IFR, or some decomposed components of PLA, to improve the char residue and char strength, which provide thermal insulation and postpone the material degradation until higher temperatures.

### 3.3. Volatile Gaseous Products of PLA Nanocomposites Analyzed by TG-FTIR

**Figure 5:**
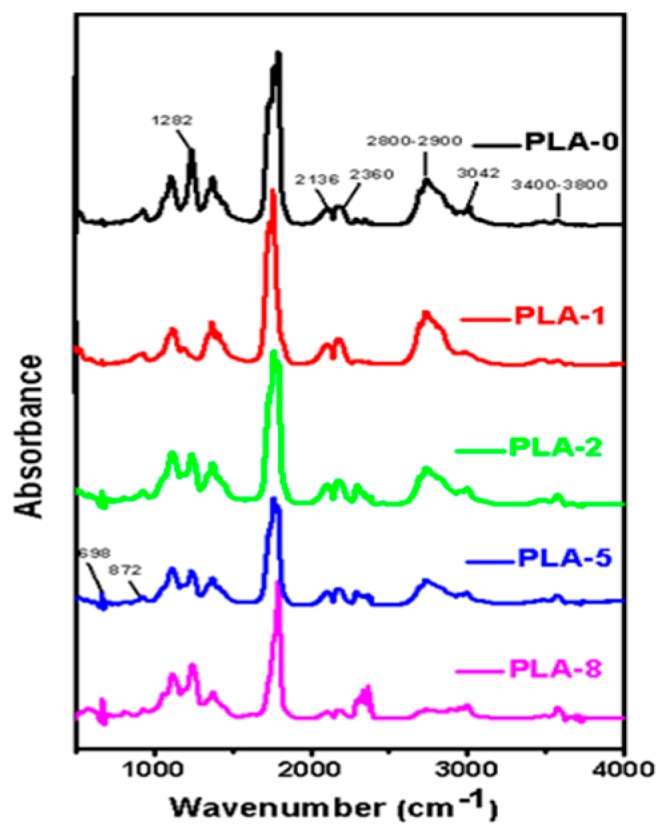
FT-IR spectra of pyrolysis products of PLA and PLA/clay nanocomposites with different surface-modified clay contents at maximum decomposition stage.

In this work, TG-FTIR was employed to investigate the effect of two OMMTs combined with IFR on the evolution of volatile gaseous components from PLA and PLA/IFR/OMMT nanocomposites during thermal degradation. The FT-IR spectra of the point of maximum weight loss are presented in Figure 5. The spectra indicate that the typical degradation process of pure PLA is almost the same as the PLA nanocomposites, except for small changes in the peak intensities of CO, CO_2_, and other peaks. However, during thermal degradation, some gaseous compounds were identified based on their characteristic absorption bands. The main components were CO_2_ (2360 cm^−1^), CO (2136 cm^−1^), water (3400–3800 cm^−1^), NH_3_ (3338, 1628, 968, and 931 cm^−1^), aromatic compounds (3042, 1618, 1508, and 698 cm^−1^), and phosphorous compounds (1282, 1086, and 872 cm^−1^) [32,33]. Additionally, to understand the changes in the evolved volume of gaseous products during heating of the PLA and PLA nanocomposites, specific components were selected and analyzed.

**Figure 6:**
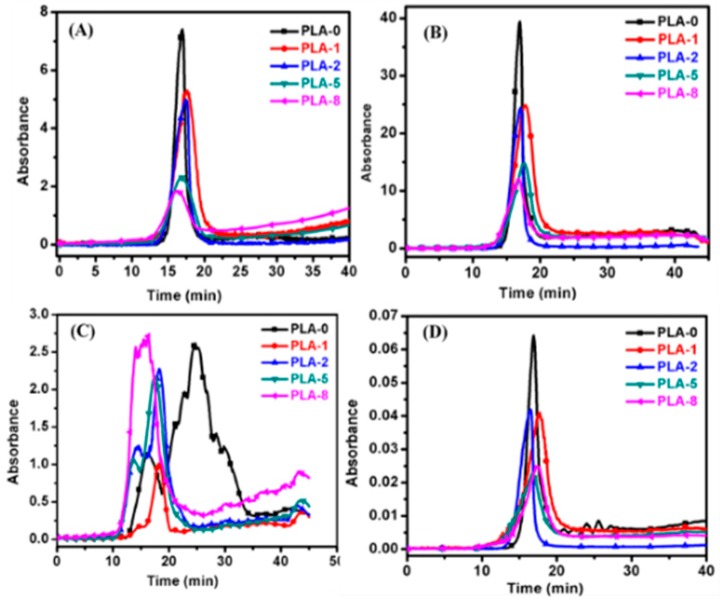
Absorbance of pyrolysis products at complete decomposition stage for PLA and PLA-clay nanocomposites. (**A**) Gram–Schmidt curves, (**B**) carbonyl compounds, (**C**) CO_2_, and (**D**) CO.

The Grams–Schmidt curves are reported in Figure 6A, showing the absorbance of the pyrolysis products with temperature. The absorption intensity of the pyrolysis products decreased from PLA to PLA nanocomposites due to the presence of OMMT improving the char strength and barrier properties. The absorbance vs temperature plots for released CO, CO_2_, and carbonyl compounds individually are shown in Figure 6B–D. For all the PLA/IFR/OMMT nanocomposites, the absorbance intensity of the released pyrolysis components was less than that of the unmodified PLA. In the presence of IFR/OMMT, acceleration of the polymer degradation to the formation of a strong char structure occurs, which can prevent further polymer degradation and evolution of combustible components. The maximum inhibition in pyrolytic volatile components, such as hydrocarbons, carbonyl compounds, and pyrolytic gases, reduces the heat release rate (HRR), total HRR (THRR), and toxic smoke. Because the volatile hydrocarbons are easily condensed and aggregate as smoke particles, a more dense smoke forms [34]. The addition of modified IFR/OMMT to the PLA subsequently decreased the volume of the released combustible gases and the weight loss, which agrees with the thermal analysis presented in the previous section. It was also observed that the presence of OMMT forms char, which can inhibit the evolution of combustible gases and toxicants. Therefore, the absorption intensities of the gaseous products from PLA-5 and PLA-8 are much less than that of PLA-0, PLA-1, and PLA-2. Moreover, evolution of gases is strongly inhibited in the OMMT-1-containing nanocomposite (PLA-5), because the presence of aromatic phosphonium compounds allows the formation of a strong graphitic crosslinked char structure at high temperatures.

### 3.4. Cone Calorimetry Analysis

**Figure 7:**
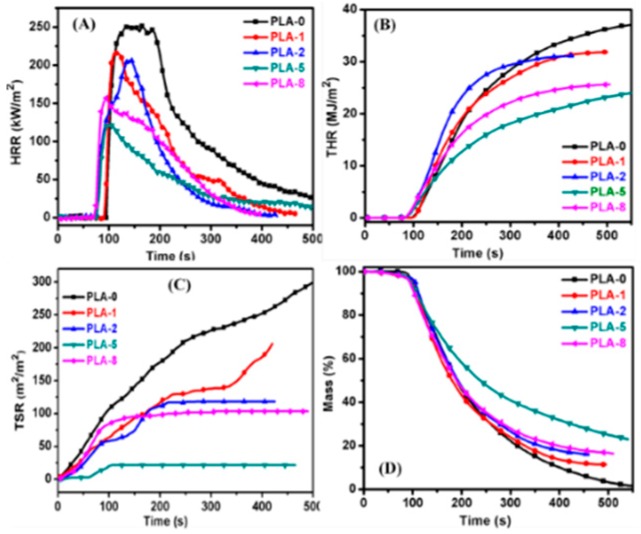
Cone colorimetry test plots of PLA/clay nanocomposites. (**A**) HRR, (**B**) THRR, (**C**) TSR, and (**D**) MLR plots.

The FR properties of PLA nanocomposites with two different surface-modified MMTs combined with IFR were studied by using cone colorimetry. The obtained results are shown in Figure 7, and the detailed data are summarized in Table 3. The data indicate that PLA-0 burns vigorously after ignition. The pHRR (peak heat release rate) and THRR (total heat release rate) values are 253.7 kW·m^−2^ and 37.2 MJ·m^−2^, respectively. After addition of IFR to the PLA, the values of TTI decreased due to pyrophosphate produced from MP during degradation, which behaves as an efficient catalyst for PLA degradation, causing it to burn quickly under heat flux. However, for the combination of modified MMT and MP-PER to the PLA, the values of TTI (time-to-ignition) further decreased because the MMT also acts as a catalyst in the polymer degradation. Subsequently, the polymer burns in early stage to forms char. The flammability properties mainly depend on the char strength [35,36].

The surface-modified MMT contains phosphonium derivatives which migrate to the polymer surface while burning and form phosphate ester crosslinked compounds with degraded PLA components. The presence of phosphorous improves the char strength and residues. Therefore, the pHRR and THR values of PLA-5 and PLA-8 are significantly lower than PLA-0. Moreover, the OMMT-1-containing PLA nanocomposites exhibited lower pHRR and THR values compared with those of OMMT-2 containing PLA nanocomposites. From Table 3, PLA-5 exhibits the lowest pHRR and THR values of 120.6 kW·m^−2^ and 25.6 MJ·m^−2^, respectively, due to highly dense char formation.

The fire growth index (FGI) and fire propagation index (FPI) are two important factors during the analysis of fire hazards [37]. The calculated FPI values are included in Table 3, which balance the fire risk between ignition and fire growth. The value of FPI for PLA-0 was, 0.4 m^2^·s/kW, representing the highest fire risk. The FPI for PLA-5 was 0.6 m^2^·s/kW, hence the fire risk was lower than PLA-0. Similarly, the FGI value indicates the flame spread rate, which decreased from 1.8 kW/(m^2^·s) for PLA-0 to 1.1 kW/(m^2^·s) for PLA-5. However, for PLA-7 and PLA-8, the FGI values slightly increased to 1.9 kW/(m^2^·s) and 1.8 kW/(m^2^·s), respectively. These FGI values increased because of the presence of unstable char formed during combustion. To completely reduce the fire hazard, the smoke release must also be reduced. Figure 7C shows the total smoke release (TSR) curves of PLA/IFR/OMMT nanocomposites. It shows that the smoke released from the OMMT containing PLA nanocomposites was strongly inhibited compared with that from unmodified PLA. As shown in Table 3, the TSR and TSP values increased from the OMMT-1-containing nanocomposites to OMMT-2-containing nanocomposites. The likely reason is that cracks in char structure caused incomplete combustion of materials. With incomplete combustion, volatile compounds emerge and condense to form smoke, resulting in increased smoke production. It can be concluded that introduction of OMMT-1 to the PLA improves the char strength and compactness during polymer burning compared with that of OMMT-2, and aromatic phosphonium derivatives on the surface-modified MMT strongly influence the fire performance of polymers due to the formation of highly crosslinked char. Also, it can be seen from Table 3 that, after the cone calorimetry test, there is no char residue left for PLA-0 (more can be found in Section 3.6). However, in the nanocomposites, the amount of char residues significantly increased to 1.3%, 11.2%, 22.9%, and 15.3% for PLA-0, PLA-1, PLA-5 and PLA-8, respectively. Plots are shown in Figure 7D, which agree with the TGA results. This indicates that with OMMTs containing PLA composites, the presence of phosphorous causes the char to become stronger and highly compact, protecting the substrate from further degradation and improving the char residue. Moreover, the presence of aromatic surface-modified MMT containing PLA nanocomposites showed high intumescent char and more compactness because of the formation of a graphitic crosslinked char structure, subsequently showing strong FR activity.

### 3.5. Analysis of Crosslinked Behavior of PLA Nanocomposites

**Figure 8:**
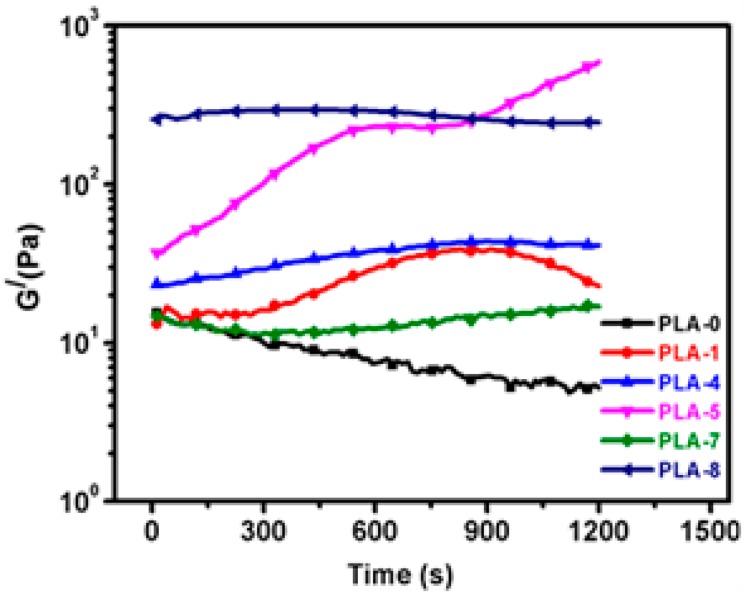
Time-dependence storage modulus (G′) neat PLA and various sweep test plots of FR PLA/clay nanocomposite samples measured at 190 °C.

Figure 8 shows plots of the storage modulus (G′) of the PLA nanocomposites as a function of time. To understand the structural alterations with time during degradation, a constant melt temperature 190 °C was used. The time-evolution of the moduli at a 1 rad/s frequency and 190 °C was studied. It is well established that the elastic modulus is more sensitive to change in molecular architecture [38].

During thermal degradation, the IFR tend to undergo cross-linking with PLA, which is clearly supported by the slight upturn in the PLA-1 curtain intervals in the viscoelastic behavior, as shown in Figure 8. The PLA and IFR may have undergone a post-condensation reaction during the constant shear rate and temperature. Further, Filippone et al. [39] reported that the oxidation of polyethylene terephthalate during time evolution exhibit both chain scission and cross-linking. Further, cross linking reactions dominate the early stage of the tests, resulting in a change of molecular structure due to the increase in the degree of cross-linking. Thus, samples exhibit a rapid growth in viscoelastic behavior. This increase in viscoelastic behavior influences the modulus of the polymer, resulting in an improvement of *G*′. For shorter times (t < 500 s), cross-linking is dominated by the chain scissoring reaction. However, the growth rate near gel formation (t ~1000 s) increases rapidly, indicating the cross-linking reaction. For nanocomposites of PLA containing IFR and OMMT (OMMT-1 and OMMT-2), only PLA-5 exhibited a crosslinking pattern, whereas the other samples showed a decrease in modulus with time, indicating absence of cross-linking of PLA. In the presence of strong interactions between the OMMT-1 and MP, more crosslinking occurs, which causes the viscoelastic parameters to continuously increase with time. Another reason may be possible synergistic effects between the OMMT-1 (right amount in the case of PLA-5) and MP, resulting in such unique behavior. Therefore, PLA-5 exhibited superior IFR activity than the other nanocomposites, with the formation of highly compact crosslinked char residue.

### 3.6. Char Residue Analysis of PLA Nanocomposites

**Figure 9:**
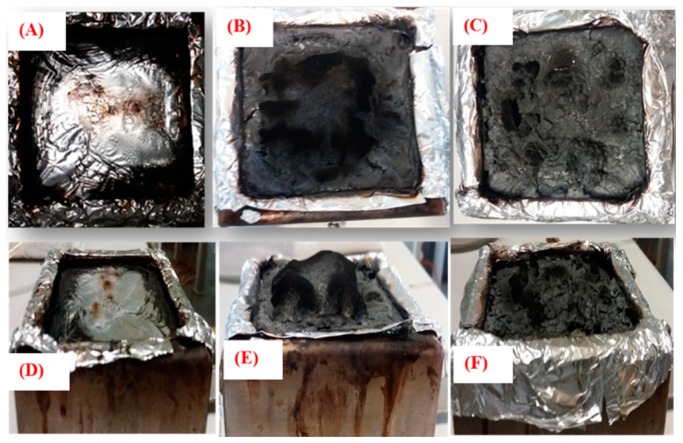
Digital photographs of burning char residues after cone calorimetry test PLA-0 (**A**,**D**), PLA-5 (**B**,**E**), and PLA-8 (**C**,**F**). 1st row for top view and 2nd row for side view.

The char morphologies of PLA and two different OMMTs containing PLA nanocomposites are shown in Figure 9. All these residual char images revealed that the flame was extinguished by the formation of intumescent char, and all the associated aspects are summarized in Table 3. PLA-0 completely burns and there is no char residue remaining, as shown in Figure 9A,D. However, the addition of OMMT to the PLA greatly improved the flammability with increasing intumescent char residues, as shown in Figure 9B,C,E,F. The char residues obtained from PLA-5 were highly intumescent compact char because less non-combustible gas escaped, resulting in greater height and strength of the char, as shown in Figure 9B,E. As we explained using SAXS and TEM analyses, in PLA-5, a greater degree of delamination of silicate layers occurs during the melt-blending process. We also believe that at this specific composition, there may be strong interactions between the OMMT-1 and MP. Hence, while burning with the clay, the high phosphorous containing compounds migrate to the polymer surface and enrich the char residue and char strength. Forming a continuous, highly compact char layer on the substrate results in the excellent FR activity of the PLA-5 nanocomposite system. Another explanation is a possible synergetic effect at this specific composition, because such an improvement in char residue was not observed in the other compositions, PLA-3 and PLA-4. Porous or loose char residues were obtained from PLA-8, as shown in Figure 9C,F, and it is clearly visible that there was less expansion and a lower height of the char. Voids and cracks in the char layer were observed, and the char was not strong enough to inhibit the evolution of volatile components. Therefore OMMT-1-containing PLA composites exhibited better FR activity than OMMT-2-containing PLA composites.

**Figure 10:**
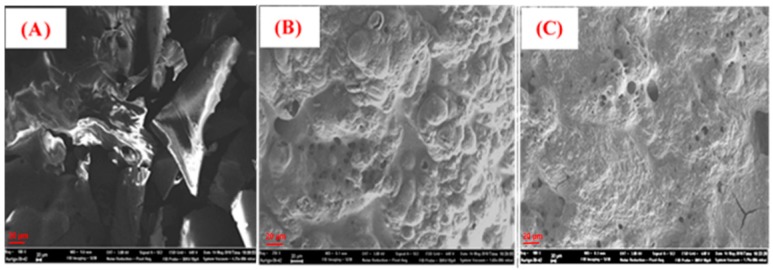
SEM images of the outer surface of char residues of PLA-0 (**A**), PLA-5 (**B**), and PLA-8 (**C**).

The SEM images of the residual char of PLA-0, PLA-5, and PLA-8 are shown in Figure 10. Figure 10A clearly shows that pieces of char layers are present. During combustion, a broken char layer and inferior char quality cannot form an effective intumescent char layer to prevent the underlying material from further degradation. Hence, serious melt drippings form and there is no char residue in the case of PLA-0. During combustion, the first stage of MP decomposition releases non-combustible components like NH_3_ and water along phosphoric acid, which can further dehydrate the polymer chains to form char as we observed in the case of nanocomposite samples. In the OMMT-1, phosphonium derivatives are formed from the surfactant during combustion at high temperatures, creating the possibility for the formation of aryl phosphine, phosphite oxides, and graphitic crosslinked char, resulting in highly compact and strong char, which inhibits the volatile components and toxic gases. The char morphology of PLA-5 is shown in Figure 10B, and it clearly exhibits a highly compact and strong char layer with bubbles, due to the release of pyrolytic components and ammonia gases accumulating inside. These chars can prevent further degradation and melt drippings. Therefore, PLA-5 displayed high FR activity with decreased HRR, THRR, and TSR. The residual char morphology of PLA-8 is shown in Figure 10C. It clearly shows thin char with some cracks and holes. The presence of aliphatic phosphorous compounds and it cannot form the graphitic strong char, hence prevent the formation of graphitic char and the char is unstable. Because of these cracks and holes in the char, volatiles easily escaped. Overall, this indicates that the presence of aromatic phosphorous derivatives contributes to the FR activity, forming a highly compact and graphitic char layer at high temperatures.

**Figure 11:**
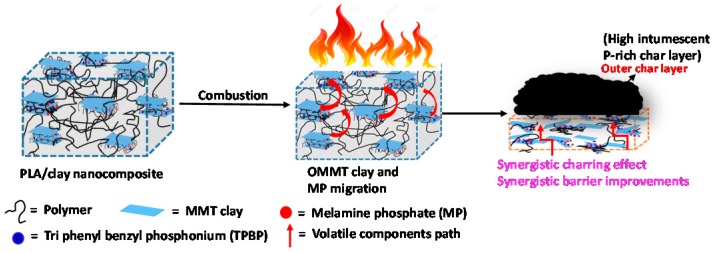
Schematic model to explain synergetic charring formation. Drawings are not to scale.

Based on the above analyses from TGA, SAXS, rheology, and SEM images of obtained residual chars after cone calorimetric test, it can be found that there is a strong synergetic effect between OMMT-1 and MP. During combustion along with MMT platelets, MP also migrates to the polymer surface, resulting in high crosslinking which can improve the viscoelastic properties of the nanocomposite. Therefore, in the case of PLA-5, a strong synergistic effect is responsible for superior FR activity which is schematically presented in Figure 11.

## 4. Conclusions

A series of FR PLA composites were developed by a melt-extrusion process from an IFR (MP/PER), pristine MMT, and various surface-modified MMTs in the PLA matrix for comparative analysis. The introduction of surface-modified MMTs along with IFR led to remarkable improvements in the charring of PLA nanocomposites, as revealed by TGA and Cone Calorimetry analyses. OMMT-1-containing PLA nanocomposites exhibited a clay composite structure with a lower percentage of intercalation and higher percentage of delaminated nanocomposites. OMMT-2-containing PLA nanocomposites showed an intercalated nanocomposite structure at all weight percentages. At 5% OMMT-1, the nanocomposite exhibited high FR activity, with low pHRR, THR, TSP, and TSR, because at this specific composition, there might be a possible synergistic effect between MP and OMMT-1. Strong intumescent FR activity with highly compact crosslinked char formation was observed. From absorption curves, it was found that all the modified MMTs containing PLA nanocomposites strongly inhibited toxicant evolution compared with that from pristine clay composites and IFR composites. OMMT-1 strongly inhibited the evolution of all the pyrolytic components. In the presence of phosphorous, the formed char was stronger, strongly inhibited combustible and toxic gas evolution, and exhibited strong FR activity. In summary, it was found that the presence of surface-modified MMTs with aromatic compounds exhibited strong FR activity due to highly cross-linked char structure formation. However, further study is needed to understand the synergistic effect between MP and OMMT-1 in the case of the PLA-5 sample.

## Figures and Tables

**Table 1:** Compositions and abbreviations of various samples used in this study.

Sample Name	Composition	PLA	IFR (MP:PER/1:1)	Pristine Clay	OMMT-1	OMMT-2
PLA-0	PLA	100	-	-	-	-
PLA-1	PLA/IFR	86	14	-	-	-
PLA-2	PLA/IFR/MMT	86	9	5%	-	-
PLA-3	PLA/IFR/OMMT-1	86	13	-	1%	-
PLA-4	PLA/IFR/OMMT-1	86	11	-	3%	-
PLA-5	PLA/IFR/OMMT-1	86	9	-	5%	-
PLA-6	PLA/IFR/OMMT-2	86	13	-	-	1%
PLA-7	PLA/IFR/OMMT-2	86	11	-	-	3%
PLA-8	PLA/IFR/OMMT-2	86	9	-	-	5%

**Table 2:** Characteristic thermal stability date for PLA and various nanocomposites calculated from TGA thermograms.

Samples	N_2_ Atmosphere				O_2_ Atmosphere			
	*T*_deg_ (°C)	*T*_Max_ (°C)	Char at 800 °C (%)	α_max_ (%/°C)	*T*_deg_ (°C)	*T*_Max_ (°C)	Char at 800 °C (%)	α_max_ (%/°C)
PLA-0	301.7	380.5	0.5	3.0	304.7	378.7	1.7	2.6
PLA-1	195.7	401.2	4.3	1.6	192.3	395.7	4.5	1.8
PLA-2	190.7	395.0	5.8	1.8	196.5	398.9	2.4	1.9
PLA-4	192.4	392.7	8.6	1.2	194.4	389.9	11.2	1.8
PLA-5	191.5	387.5	9.9	1.0	193.5	381.5	18.2	1.0
PLA-7	194.7	383.6	7.1	1.8	191.3	378.5	2.6	2.1
PLA-8	193.5	371.4	10.4	1.0, 0.0	190.8	379.8	6.0	1.0, 0.8

PLA-0, neat PLA; PLA-1, PLA/IFR blend; PLA-2, PLA/IFR/MMT composite; PLA-4, PLA/IFR/OMMT-1 (3 wt.%) nanocomposite; PLA-5, PLA/IFR/OMMT-1 (5 wt.%) nanocomposite; PLA-7, PLA/IFR/OMMT-2 (3 wt.%) nanocomposite; PLA-8, PLA/IFR/OMMT-2 (5 wt.%) nanocomposite.

**Table 3:** Cone colorimetry data of neat PLA and various nanocomposites.

Samples	pHRR (kW·m^−2^)	TTI (s)	t_PHRR_ (s)	THR (MJ·m^−2^)	FGI (kW/(m^2^·s)	FPI (m^2^s/kW)	TSR (m^2^/m^2^)	TSP (m^2^)	Residue (wt.%)
PLA-0	253.7	92.7	142.2	37.2	1.8	0.4	295.9	37.3	1.3
PLA-1	216.7	88.6	126.9	31.9	1.7	0.4	204.9	31.9	11.2
PLA-2	205.5	72.5	139.1	31.1	1.5	0.4	118.2	31.2	16.2
PLA-3	161.2	75.9	121.9	28.1	1.3	0.5	24.1	27.0	11.2
PLA-4	154.2	76.7	118.2	26.9	1.3	0.5	21.1	26.5	16.8
PLA-5	120.6	73.2	109.5	25.6	1.1	0.6	09.4	21.4	22.9
PLA-6	181.7	77.0	115.6	26.5	1.6	0.4	125.6	29.8	09.8
PLA-7	164.1	70.9	84.1	25.9	1.9	0.4	98.3	25.1	13.8
PLA-8	163.4	69.9	89.4	24.0	1.8	0.4	102.4	27.5	15.3

pHRR, peak heat release rate; TTI, time-to-ignition; HRR, heat release rate; THR, total heat release; TSR, total smoke release; MLR, mass loss rate. (FGI = pHRR/tPHRR and FPI = TTI/pHRR).
